# Typho-Malarial Fever

**Published:** 1882-12-20

**Authors:** 


### *TYPHO-MALARIAL FEVER.*

A subscriber requests that something be put in the JOURNAL on the subject of Typho-Malarial Fever. We trust some one of our readers, who has had experience in the treatment of the disease, will comply with this request and write us a practical article on the subject.

There are those who claim that there is no distinct disease to which the appellation, Typho-Malarial, can be given. To such we state that we have seen many cases of an affection to which the name is eminently applicable. It might be termed a form of intermittent fever which does not yield to quinine. There are often well marked morning intermissions, especially in the outset of the attacks. Sometimes a decided remission, in which the thermometer will indicate a temperature scarcely to be called febrile, and which in the afternoon will run up to 103°-104°. Quinine does not cut short the fever, and, indeed, makes little or no impression upon it.

The nose bleed, so common in the early stages of typhoid fever, is often found, but there is very little tendency to diarrhœa or tympanitis. The patient usually has considerable appetite. At evening the febrile excitement is often high, and there is not unfrequently some degree of delirium, but not the low muttering delirium of regular typhoid. Sordes is not seen upon the teeth as in typhoid fever.

In our experience these cases usually continue two weeks. The plan of treatment which we have adopted, and which has been successful, is as follows: We give quinine in most cases to the extent of 8 to 12 grs. in the forenoon, as we think it lowers the temperature and supports the patient, and under its influence the tendency of the fever to decline on the septinary or seven day periods is encouraged.

In the afternoon we give a teaspoonful of a solution of aconite (the strength of 10 drops in a glass of water) every hour during the height of the exascerbation, and four times a day we give as an antiseptic, and as prophylactic to the possibility of inflammation or ulceration in Peyers glands, the syrup of tar in teaspoonful doses every four hours. This is substituted for the spirits of turpentine, usually relied upon in typhoid affections, as being more pleasant and less likely to nauseate the stomach or to produce strangury. Frequently we combine it with eucalyptus as follows:

℞ Syrup of tar.............................................℥ iij,

Tinc. eucalyptus.........................................ʒ ij.

M. Shake and give teaspoonful four times a day. The eucalyptus is also antiseptic, and while resembling the terebinthenates in property, is also supposed to be anti-malarial. The dose appears small in this prescription, but in larger doses it will be found to produce a sensation of fulness in the head, with a degree of drowsiness which, if long continued, might lead to delirium or cerebral complication.

Purgatives are to be given with caution in this disease. Though diarrhœa is not commonly found, the bowels are easily moved, and usually there is a spontaneous evacuation almost daily. At the outset, it is well to give a dose of castor oil, but it is rarely necessary to give a purgative during the subsequent attack. Occasionally we find it advantageous to evacuate the bowels with an enema, or with a mild laxative—as the syrup of rhubarb. This, with application of mustard in case of local pain, and appropriate treatment to any other indication that may present during the attack, has been the plan we have pursued in the treatment of this affection. W.

